# Tailoring Therapeutic Responses via Engineering Microenvironments with a Novel Synthetic Fluid Gel

**DOI:** 10.1002/adhm.202100622

**Published:** 2021-06-23

**Authors:** Nicola C. Foster, Piers Allen, Alicia J. El Haj, Liam M. Grover, Richard J. A. Moakes

**Affiliations:** ^1^ Healthcare Technologies Institute University of Birmingham Birmingham B15 2TT UK

**Keywords:** cell scaffolds, fluid gels, functionalization, PEG, soft‐particle glasses

## Abstract

This study reports the first fully synthetic fluid gel (SyMGels) using a simple poly(ethylene glycol) polymer. Fluid gels are an interesting class of materials: structured during gelation via shear‐confinement to form microparticulate suspensions, through a bottom‐up approach. Structuring in this way, when compared to first forming a gel and subsequently breaking it down, results in the formation of a particulate dispersion with particles “grown” in the shear flow. Resultantly, systems form a complex microstructure, where gelled particles concentrate remaining non‐gelled polymer within the continuous phase, creating an amorphous‐like interstitial phase. As such, these materials demonstrate mechanical characteristics typical of colloidal glasses, presenting solid‐like behaviors at rest with defined yielding; likely through intrinsic particle‐particle and particle‐polymer interactions. To date, fluid gels have been fabricated using polysaccharides with relatively complex chemistries, making further modifications challenging. SyMGels are easily functionalised, using simple click‐chemistry. This chemical flexibility, allows the creation of microenvironments with discrete biological decoration. Cellular control is demonstrated using MSC (mesenchymal stem cells)/chondrocytes and enables the regulation of key biomarkers such as aggrecan and *SOX9*. These potential therapeutic platforms demonstrate an important advancement in the biomaterial field, underpinning the mechanisms which drive their mechanical properties, and providing a versatile delivery system for advanced therapeutics.

## Graphical abstract

Novel synthetic‐based fluid gels (SyMGels) are developed and studied as a platform technology to create bespoke cellular microenvironments. Controlled gelation within a sheared environment promotes a complex microstructure, providing highly tuneable behaviors akin to soft glasses: engineering materials ideal for dynamic/articulated anatomical regions. The chemically “simple” polymers allow for ease of customization/functionalization, using biomolecules as decoration, to influence cell responses.

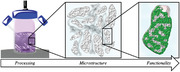

## 1 Introduction

The role of biomaterials within medicine and tissue engineering has received growing attention within recent years, although their usage has been documented for millennia.^[^ ^1^ ^]^ Scientific and technological advances have led to vast improvements within healthcare, stimulating the next generation of increasingly more complex materials.^[^ ^2^, ^3^ ^]^ One such leap was demonstrated in the 1930s, where advances in synthetic polymers led to applications ranging throughout the human anatomy: cardiovascular, orthopaedics, ophthalmic, dental and neural.^[^ ^4^ ^]^ These polymeric scaffolds provided replacement of the “inert” standards of care, with no inherent regenerative properties, with those capable of, for example, stimulating osteoconductive mechanisms.^[^ ^5^ ^]^ The principle of providing scaffolds for cell infiltration and native healing processes is not limited to bone. Many soft tissue applications have been addressed through the use of synthetic scaffolds, however, the complex nature of many diseases still demand better integration and/or functionality combined with suitable delivery approaches. Decellularized tissue scaffolds have been proposed to address structural and biological cues, as the extracellular matrix (ECM) provides a direct mimetic of the native environment.^[^ ^6^, ^7^ ^]^ Although theoretically ideal and currently used in the clinic for multiple applications, the requirement for harsh chemical processing, potential variation between batches and patient rejection,^[^ ^8^ ^]^ have hindered large scale adoption. Hydrogels have been regarded as an alternative for such materials, providing an ECM‐like structure to immobilise cells for transplantation.^[^ ^9^ ^]^ Their tolerance in biological environments, high‐water content, mass transport and versatility have directly resulted in such materials becoming adopted into numerous tissue engineering and drug delivery applications.^[^ ^10^ ^]^ However, translation of these new materials is still slow, with large costs surrounding toxicology studies stemming from chemical, physical and morphological roles in modulating cellular events.^[^ ^11^ ^]^

One means of navigating the high costs and risks posed by the translation processes is through the use of currently approved materials, and re‐structuring them through a microstructural design process.^[^ ^12^ ^]^ Commonly used throughout many industries, the microstructural design approach to formulation engineering focuses on the interplay between three key areas: raw materials, processing and material properties.^[^ ^13^ ^]^ Again, hydrogels have lent themselves to such approaches,^[^ ^14^, ^15^ ^]^ where careful control over chemical properties such as polymer concentration, chain length, chemical backbone (hydrophobic/hydrophilic balance), charge and branching,^[^ ^16^ ^]^ and processing parameters, degree of curing/gelation,^[^ ^17^ ^]^ allow for the manipulation of material behaviors including strength, elasticity and yielding. This technique can be employed to materials commonly found within the medical field (PGLA, PEG, alginate, etc.) using processing strategies (mixing, drying, heating, etc.), or specific chemistries (phase separation, pH, electro‐potential), to fabricate materials with increasingly complex mechanical behaviors.^[^ ^18^ ^]^ Indeed, granular hydrogels, where the formation of microgel particles can be facilitated through either templating (phase separation techniques such as emulsions and microfluidic devices) or shear break down of a pre‐formed gels, are key examples of using processing to influence bulk mechanical properties.^[^ ^19^, ^20^ ^]^

Fluid gels, again microgel suspensions, use a bottom‐up approach to controlling particle formation; “grown” through a nucleation and growth mechanism, which is ultimately controlled by shear imparted on the system.^[^ ^21^ ^]^ Gelation kinetics therefore play a pivotal role during the fabrication process, with resulting suspensions determined by the equilibrium between gel growth (gelation kinetics) and extent of mixing (timescale of shearing, shear rate).^[^ ^22^ ^]^ By controlling microgel formation in this way, it is possible to engineering rheological properties, arising through weak interactions at the particle interfaces, where complete gelation has not been allowed to occur.^[^ ^23^, ^24^, ^25^, ^26^ ^]^ Ultimately, these densely packed gelled particles have the ability to “squeeze” past each other under large strains, providing a pseudo‐solid behavior at rest with prominent shear thinning capacities.^[^ ^26^, ^27^, ^28^ ^]^ Apart from their rheological characteristics, their one‐step manufacturing process and compatibility with regulator‐approved materials, has likely driven their application within the translational field, providing a highly dynamic platform for a range of therapies: leading to enhanced retention and delivery in ocular applications;^[^ ^29^ ^]^ keratinocyte delivery for burn wounds;^[^ ^30^ ^]^ and, determination of cell fate through controlled geometric constraint.^[^ ^31^ ^]^ Although the versatility of such materials has been demonstrated, they are still limited within highly complex healing pathways for example, angiogenesis, where multi‐stage mechanisms require delivery of specific actives at different timescales.^[^ ^32^ ^]^ A proposed method of obtaining such defined release profiles are through programmed delivery techniques, whereby chemically sensitive bonding is used to retain therapeutics on the gel, releasing once stimulated by a biological cue.^[^ ^33^ ^]^ Although functionalization of polysaccharide‐based materials is achievable,^[^ ^34^, ^35^, ^36^ ^]^ chemical alteration is typically undertaken prior to gelation, accompanied by complicated synthesis and at risk to affecting gelation properties thereafter.^[^ ^37^ ^]^ As such, the often‐complex chemical structures (in terms of both chemical moieties and disparity in repeating saccharide units) of the polysaccharides currently used to formulate fluid gels, do not lend themselves to easily facilitate chemical tethers between microgel particle and biologically active molecules. Thus, to date, fluid gels have not enabled highly modulated therapeutic delivery systems, instead relying heavily on diffusion mechanisms to mediate payload release.

This study, reports a fluid gel formed using with a synthetic polymer, poly(ethylene glycol); due to its clinical relevance, being commonly used in CE marked medical devices. In this way the resultant materials are open to further functionalization, through the addition of pendant groups, small molecules or proteins, which can be used to direct cell response. As such, PEG‐based fluid gels (SyMGels – microgel suspensions specifically fabricated through a bottom‐up approach), were prepared and characterised for their mechanical characteristics. Cell behaviors were evaluated in 2D culture, pre‐ and post‐functionalization with a range of biologically relevant macro‐molecules, in order to demonstrate the capabilities for control over the cellular microenvironment. Moreover, functionalized‐SyMGel's potential to drive healing was investigated in vitro, specifically focused on field of cartilage therapy: aiming to enhance current clinical practises in cell therapy,^[^ ^38^ ^]^ through effective delivery/creation of a pro‐healing environments, to help improve and ensure engraftment.

## 2 Results

### 2.1 Fabrication of Microgel Suspensions

**Figure 1:**
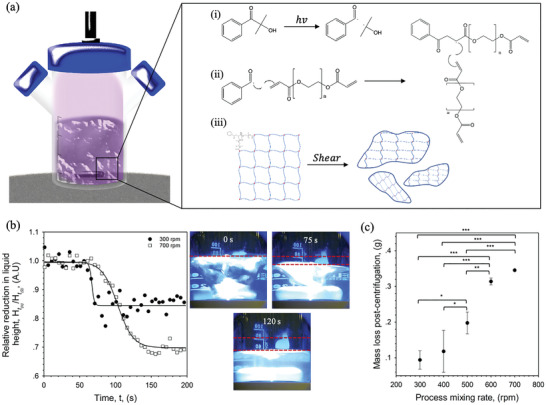
Formation of synthetic microgel suspensions (SyMGels) via sheared gelation. a) Schematic representation for the fabrication of SyMGels, with proposed gelation mechanism: i) radical formation; ii) initiation and propagation, and; iii) restriction of gel growth through applied shear to form terminated particles. b) “Gelation” profiles for SyMGels prepared at mixing rate of either 300 or 700 rpm. Profiles were obtained by measuring the deviation from initial liquid height as a function of time as shown by the photographic representations at 0, 75, and 120 s (700 rpm). c) Determination of the gelled phase using centrifugation. Mass of continuous phase removed as a function of processing mixing rate from a 0.5 g aliquot after centrifugation at 17000 rcf for 10 min. All data presented is the average on *n* = 3, with error bars demonstrating the 95% confidence interval. Statistical analysis was conducted using one‐way ANOVA with significance denoted as * *p* < 0.05, ** *p* < 0.01, and *** *p* < 0.001.

Synthetic microgel suspensions (SyMGels) were prepared using a shear‐gel technique previously reported for polysaccharide based fluid gels;^[^ ^12^, ^21^, ^31^, ^39^, ^40^ ^]^ whereby shear was applied to a polymer sol undergoing a sol‐gel transition. This process has been depicted in **Figure** **1**a, highlighting the use of UV light to stimulate the formation of radicals (Figure 1ai) which promote free radical polymerisation, propagating through the carbonyl species within the acrylate groups (Figure 1aii). However, unlike typical polymerisation processes, growth termination is controlled by the presence of shear, resulting in a particulate suspension instead of a single continuous network (Figure 1aiii).

Building on previously reported data showing a direct correlation between particle formation and viscosity increase,^[^ ^21^, ^28^, ^41^ ^]^ the reduction in vortex (liquid height), as result of thickening, was used as a qualitative means to measure gelation (Figure 1b). It was observed that all systems, irrespective of the mixing applied, started to gel (defined as the onset of change) after the same length of irradiation, ca. 65 s, undergoing a decrease in vortex height until a secondary plateau/equilibrium was reached. Comparison of the profiles highlighted a shift in the “rate of gelation”, with higher mixing speeds resulting in slower curing.

**Figure 2:**
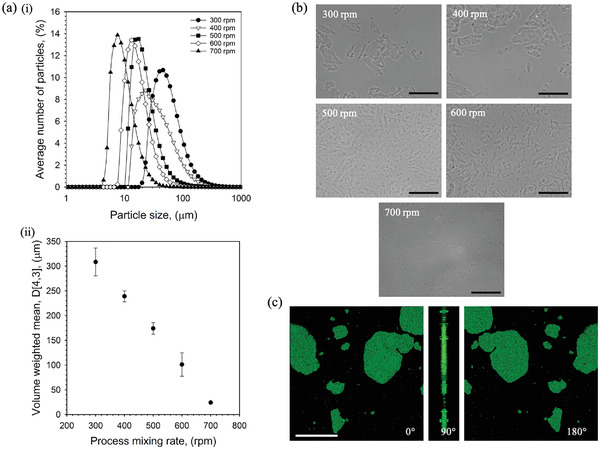
Synthetic microgel particle shape and size. a) static light scattering data for SyMGels prepared at various mixing rates. i) Particle size distributions as a function of processing mixing rate and ii) volume weighted averages (D[4,3]) taken from the distributions showing a decreasing linear trend in size as a function of applied mixing. b) Optical micrographs obtained using phase contrast microscopy for diluted (1:4) SyMGel systems prepared at varying mixing rates. c) 3D stacked CSLM images rotated through 90° and 180° to show particle thickness (particles prepared at 300 rpm). Gelled particles were stained with Rhodamine 6G and images using a 543 nm laser. (Scale bars represent 100 µm). All data presented is the average on *n* = 3, with error bars demonstrating the 95% confidence interval.

The extent of gelation was further probed using centrifugation to separate the gelled and continuous phases (Figure 1c). Increasing the shear applied during processing resulted in a linear increase (*p* < 0.01) in extractable continuous phase, highlighting a reduction in the degree of gelled particles being formed throughout curing. The effect of mixing on both particle size and morphology has been shown in **Figure** **2**. A static light scattering technique was used to determine size distributions for all SyMGel systems (Figure 2ai). In all cases, SyMGels showed a broad monomodal peak, suggesting a range of particle sizes. Average particle size, D[4,3] values, were determined to show average changes in particle size (Figure 2aii). Again, a linear relationship between the applied mixing and resultant particle size was clearly observed, forming larger particles at low mixing rates (308±28 µm at 300 rpm) and smaller particles at higher processing rates (24±0.8 µm at 700 rpm). Particle micrographs cohere with sizing data, showing a reduction in size as a function of the mixing applied (Figure 2b). Particle morphology appeared to increase in uniformity as the mixing was increased, characterised by a higher length to width ratio. More in‐depth analysis of the particle structure, using confocal microscopy, highlighted a relatively thin structure to the particles, represented by a “disc‐like” shape ca. 10 µm in thickness (Figure 2c).

### 2.2 Suspension Material Properties

**Figure 3:**
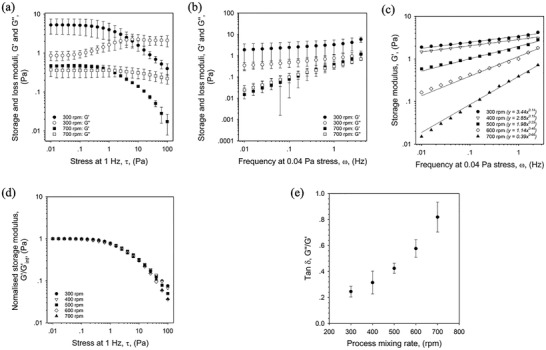
Mechanical behavior of SyMGel systems. a) Amplitude sweep, stress controlled, for SyMGel suspensions prepared at either 300 or 700 rpm. b) Frequency sweeps obtained at 0.04 Pa stress for SyMGels prepared at 300 and 700 rpm. c) Storage moduli (*G*′) obtained via frequency sweeps (0.04 Pa stress), as a function of processing mixing rate. Lines of best fit added to each data set with equation of the line shown in the legend. d) Collapsed amplitude sweeps for SyMGels prepared at varying processing mixing rates. e) Change in Tan*δ* as a function of the processing mixing rate used during SyMGel curing. All data presented is the average on *n* = 3, with error bars demonstrating the 95% confidence interval.

**Figure 4:**
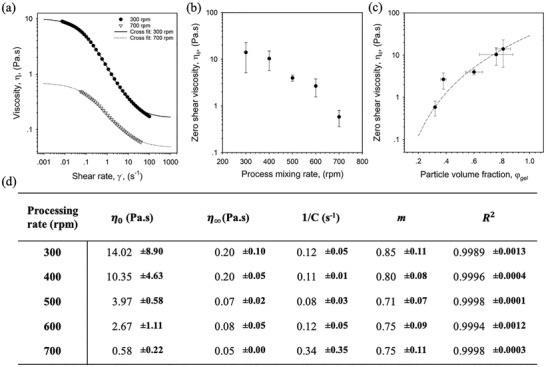
Flow behavior of the SyMGel suspensions. a) Shear rate ramps for SyMGels prepared at mixing rates of 300 and 700 rpm obtained over a 1 min sweep. Fit to the Cross model applied and used to determine data presented in the table below it. b) Zero shear viscosity (*η*_0_) data obtained using the Cross fit plotted as a function of processing rate. c) Zero shear viscosity plotted as a function of the particle volume fraction (*φ*_gel_) determined using the centrifugation data presented in Figure 1c. Fit to Mark‐Houwink equation for concentrated systems (*M* > *M*_c_), *η*_0_ = *K_T_* *M* ^3.4^, where *K*_T_ was used as a fitting factor and *M* has been replaced by the particle volume fraction, *φ*_gel_. d) Table of data collated from fitting flow profiles to the Cross model where, *η*_0_ is the zero‐shear viscosity, *η*_∞_ is the infinite shear viscosity, 1/*C* is the critical shear value to induce thinning, m is the thinning index, and R^2^ is the statistical measure of fitting. All data presented is the average on *n* = 3, with error bars demonstrating the 95% confidence interval.

SyMGel viscoelasticity was studied under both small (**Figure** **3**) and large deformation (**Figure** **4**). Linear rheology was used to understand SyMGel behavior at rest. At 1 Hz, all SyMGel systems were characterised by a storage modulus (*G*′) that dominated the loss modulus (*G*″). Increasing the stress applied to the systems resulted in a shift out of an equilibrated state (LVeR), to the extent that a cross‐over to a loss dominated system (*G*″ > *G*′) occurred (Figure 3a). Mechanical spectra for the storage moduli, under increasing stress were collapsed to show superimposablity, with all curves displaying the same LVeR and reduction in *G*′ thereafter (Figure 3d). Frequency dependent data obtained at a stress within the LVeR for all systems highlighted that SyMGels prepared at the highest mixing rate, 700 rpm, behaved as a viscoelastic liquid initially dominated by the loss modulus and crossing‐over at higher frequencies to *G*′ dominance. However, for all other systems, across the full frequency range studied (0.01–10 Hz), *G*′ dominated *G*″, suggesting gel‐like behaviors. Gel strength was characterised by both magnitude of *G*′ and loss tangent (tan*δ*). All systems showed weak gel‐like behaviors exhibiting values for tan*δ* ranging between 0.2 and 0.9 (Figure 3e), with spectra showing varying frequency dependencies. Frequency dependence was quantified by applying a fit to the data and comparing the power indices (Figure 3c), showing less of a dependency, 0.14, for systems prepared at 300 rpm increasing to 0.15, 0.29, 0.46, and 0.66 for systems prepared at 400, 500, 600, and 700 rpm, respectively.

Non‐linear rheology was used to better understand the materials ability to flow at large strains (for example, if injected). All systems showed highly shear thinning suspensions which could be closely fitted to the Cross model (Figure 4a), providing a means of comparing between systems. Data obtained from fits to the Cross model have been presented in the table in Figure 4d, showing similar values for the critical shear rate required to induce flow (1/*C*) and thinning index (*m*) for all systems; correlating closely to the amplitude data presented in Figure 3d. Changes in zero shear viscosity (*η*_0_) were plotted as a function of processing rate (Figure 4b) and particle volume fraction (*φ*_gel_) (Figure 4c), in order to understand the extent that material viscosity was dependent on processing.

*η*_0_ correlated closely with data collected for gel strength, decreasing from 14.02±8.9 Pa s at 300 rpm to 0.6±0.2 Pa s at 700rpm, suggesting the resulting volume occupied by the particles plays a key role in system rheology. Data collected for the degree of gelation (Figure 1c) was therefore used to determine particle volume factions (*φ*_gel_), assuming the density of the supernatant removed to be that of PBS (1.065 g cm^−3^). In order to probe material behavior further, *η*_0_ was fitted to a model proposed for concentrated flexible linear polymers solutions (Equation 1), substituting the term for polymer length with the volume occupied by the gelled particles, *φ*_gel_

(1) $$\eta_{0}=K_{T}\;M^{3.4}$$

where *K*_T_ was used as a fitting factor and M has been replaced by the particle volume fraction, *φ*_gel_. Correlation to the model suggests that SyMGels behave similarly to concentrated polymer solutions, where decreasing the mixing rate throughout processing results in a more “highly concentrated” solution; with particles likely providing an excluded volume effect.

### 2.3 Functionalization of SyMGels to Control Cellular Microenvironments

**Figure 5:**
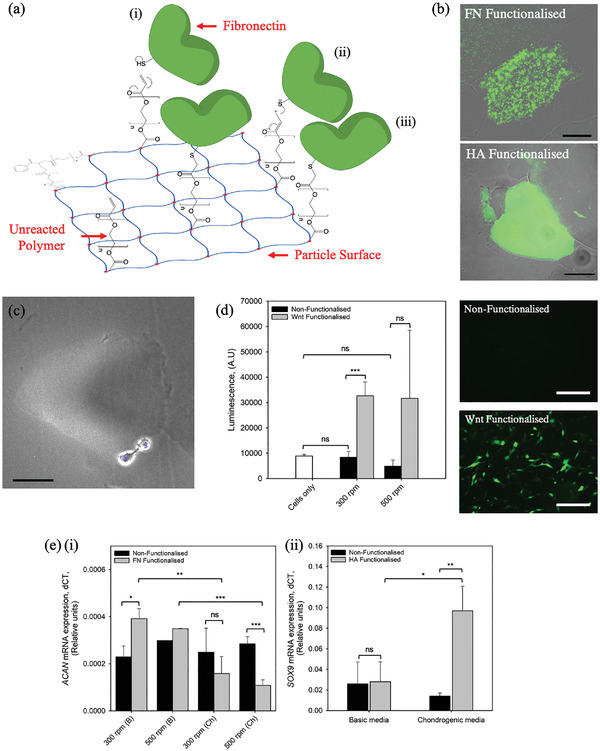
Controlling cellular microenvironments through functionalization. a) Schematic showing the proposed mechanism for the functionalization of SyMGel particles with bioactive molecules and ECM components. Mechanism is based on a typical Michael‐type reaction, reactions steps from reactants to product are highlighted in (i) through to (iii). b) Optical micrographs with fluorescent overlays of SyMGel particles (prepared at 300 rpm) functionalized with fibronectin (FN) and hyaluronic acid (HA). (Scale bar represents 100 µm). c) Micrograph of a FN‐SyMGel particle (prepared at 300 rpm) with chondrocytes adhered to the surface. (Scale bars represent 100 µm). d) Wnt‐SyMGel activation using a luciferase reporter line. Micrographs visually shows activation of the cells via fluorescence. (scale bar represents 150 µm). e) Control over key chondrocytic markers using, i) FN‐SyMGel and ii) HA‐SyMGel (300 rpm) microenvironments. All data presented is the average on *n* = 3, with error bars demonstrating the 95% confidence interval. Statistical analysis was conducted using two‐way ANOVA with significance denoted as * *p* < 0.05, ** *p* < 0.01 and *** *p* < 0.001.

Functionalization of the particles with various bio‐macromolecules was achieved via a Michael‐type reaction between the thiol groups (present within the cysteine residues of the proteins, fibronectin and Wnt‐3a, and grafted onto hyaluronic acid) and the unreacted carbonyl groups on the particle surface;^[^ ^42^ ^]^ found at the polymer terminating ends and gel junction zones (**Figure** **5**a). Bonding of the protein to the particle surface was determined using immunohistochemical and fluorescent protein binding. Micrographs showed localisation of both the fibronectin and hyaluronic acid to the surface of the particle (Figure 5b), however, coverage was not homogenous across all particles, with some particles remaining un‐coated across all systems. Moreover, when functionalized with the fibronectin, cells were seen to attach to the particle interface (Figure 5c), resulting in morphological changes towards a more spheroidal nature. Again, cell adhesion was not observed across all particles.

**Figure 6:**
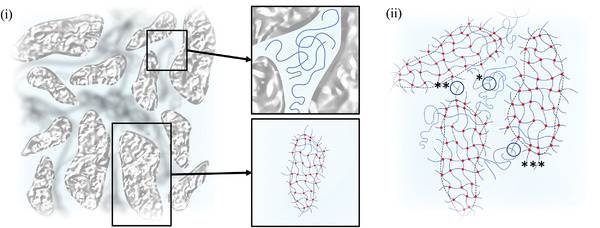
Complex fluid gel microstructure. Schematic diagrams of the complex fluid gel microstructure showing i) the system at the macroscale (inserts used to better depict the amorphous‐like interstitial phase and particle structures). ii) Diagram showing the various system interactions: * polymer‐polymer entanglements in the interstitial phase; ** particle‐particle interactions when in close proximity; *** particle‐polymer interactions formed at the particle interface and interstitial layers.

The ability to manipulate cellular microenvironments, through specific decoration of the SyMGel particles was studied using both reporter cells and quantitative polymerase chain reaction (qPCR). Wnt‐3a activation of cells was determined using a luciferase assay (Figure 5d). In the presence on the SyMGels only, no change in cell activation, as determined by fluorescence intensity, was observed; confirmed by fluorescent microscopy. However, once functionalized (Wnt‐SyMGel), a statistically significant (*p* = 0.001) increase in fluorescence was demonstrated, signifying both attachment of the protein, and more importantly retained activity. Fibronectin‐ (FN‐SyMGel) and hyaluronic acid‐ (HA‐SyMGel) functionalized systems demonstrated the ability to regulate both aggrecan (*ACAN*) and *SOX9* expression, respectively (Figure 5e). Here, expression was shown to be dependent on both the decoration of the particles and the culture medium. Non‐functionalized SyMGels, irrespective of media type (basic or chondrogenic), demonstrated no change in either the *ACAN* or *SOX9* gene. However, once functionalized, FN‐SyMGels (Figure 5ei), showed the ability to both upregulate *ACAN* (300 rpm samples, *p* < 0.05) in the presence of basic media; or, down regulate the same gene (500 rpm samples, *p* < 0.001) when cultured in chondrogenic media. HA‐SyMGels (Figure 5eii) showed similar results for the expression of *SOX9*, showing a significant (*p* < 0.01) increase in the presence of chondrogenic media.

## 3 Discussion

Over recent years the need for new materials which can aid regenerative medicine processes has become increasingly more apparent. One class of materials which, due to their unique material behaviors, have gained much attention are fluid gels. Fluid gels, like their quiescently gelled counterparts, possess all of the widely‐reported advantages of hydrogels (high water‐holding capacities, biocompatibility, ECM‐like structures, good mass transport), yet they also offer the ability to control the microstructure throughout the gelation process into a particulate suspension, which results in solid‐like behavior at rest and liquid‐like behavior on shearing.^[^ ^25^ ^]^ This has provided a dynamic platform, that can be easily applied as a liquid, rapidly re‐structuring in situ, forming a solid‐like material with high retention properties. Thus, such systems can be employed across a wide variety of applications unsuited to more conventional hydrogels including: therapeutic ocular delivery, cell spraying and 3D printing.^[^ ^12^, ^29^, ^30^, ^31^, ^40^, ^43^ ^]^ To date, these materials have only been demonstrated using polysaccharides as the gelling polymer, which despite offering numerous advantages compared to synthetic polymers, are chemically limited with regard to the addition of functional groups/co‐polymerisation (both previously shown to provide versatile delivery and breakdown mechanisms).^[^ ^1^, ^44^, ^45^, ^46^, ^47^ ^]^

This study demonstrated the first fabrication of synthetic polymer fluid gels, using photo‐polymerisation to undertake gelation; resulting in a suspension of discrete gelled particles, henceforth termed SyMGels (Synthetic MicroGel suspensions). The formation of these gels followed a typical free radical polymerisation process, whereby junction zones were formed via radical‐induced bonds between the acrylate groups at the end of the PEGDA chains.^[^ ^48^ ^]^ Increases in polymer chain length during propagation resulted in polymer precipitation, phase separation and the formation of water‐in‐water gelled nuclei.^[^ ^49^ ^]^ Interfacial polymerisation from the surrounding polymer provides growth of the nucleated regions, undergoing the Trommsdorff‐Norrish effect, and finally the formation of anisotropic particles. Changes in viscosity accompanying auto‐acceleration, as the gelled entities changed the effective phase volume of the system (trapping of the continuous phase),^[^ ^50^, ^51^ ^]^ coupled with gel shrinkage^[^ ^52^ ^]^ were used as the basis to qualitatively assess and compare the gelation process; by measuring changes in sol volume throughout processing. Data collected suggested the ability to control the kinetics of suspension formation. Although there was little change in the lag time prior to gelation, increased mixing slowed the curing process, augmenting the time required to reach an equilibrium between both gelling and shearing processes. Ultimately, termination was hindered by two factors, the increase in system viscosity and the applied mixing. As such, particle formation could take two routes: 1) formation of particles in the shear flow, or, 2) rapid formation of gel particles and subsequent break down.^[^ ^22^, ^53^ ^]^ However, in both cases size is dictated by the shear/turbulent flow of the processing unit,^[^ ^21^ ^]^ with particle morphology subsequent to the energetically more favourable gelation in line with shear‐flow.^[^ ^28^ ^]^ It is therefore understandable that increased mixing (shear separation) during gelation resulted in a lower degree of particle formation, increasing residual non‐gelled polymer.

The interplay between material behavior and microstructure has long been established with many materials shown to be a function of intrinsic properties such as particle size, shape, crosslink density, etc. SyMGels demonstrated highly tuneable rheological properties as a function of a single processing parameter, mixing speed. Dynamic stress sweeps identified three key regions: linear region (*σ* > *σ*_c_); the onset of visco‐plastic yielding (*s*_c_ < *s*_y_ < *s*_f_); and, complete fluidisation (*s* > *s*_f_). Interestingly, although the magnitude of the storage modulus (*G*′) changed depending of the degree of mixing applied during fabrication, once collapsed, all plots were identical, suggesting a similar mechanism for suspension stability and breakdown. Such inferences were reiterated in the large deformation data, with flow profiles fitted to the Cross model, highlighting similarities across critical flow values (1/*C*) and thinning indices (*m*). Comparison of the zero shear viscosity (*η*_0_) as a function of the particle volume fraction (*φ*_gel_) provided a fit to the Mark‐Houwink equation for concentrated flexible linear polymer chains.^[^ ^54^ ^]^ It is suggested such behaviors arise from the unreacted polymer at the particle surface and non‐gelled PEGDA, forming a secondary non‐gelled interstitial phase; this phase then becomes increasingly concentrated with *φ*_gel_ as the particles effectively trap the continuous aqueous phase (**Figure** **6**). To this end systems are analogous to a semi‐crystalline matrix surrounded by amorphous polymer, similar to systems described for soft‐particle and colloidal glasses.^[^ ^55^, ^56^, ^57^, ^58^, ^59^ ^]^ As such, SyMGel small deformation rheology can be described in the same way,^[^ ^58^ ^]^ behaving as a solid at low stresses/strains, whilst shear thinning in their non‐linear regimes exhibiting liquid‐like behavior.^[^ ^55^, ^60^ ^]^ As *φ*_gel_ increases the system becomes increasingly confined, resulting in systems where Brownian motion is no longer possible, leading to dynamic arrest, as particles form “cages” that sterically prevent movement,^[^ ^55^, ^56^ ^]^ until a jammed system is reached (typically at *φ* ca. 0.83).^[^ ^61^ ^]^ Such observations are mirrored in the oscillatory data, with particles formed at lower processing rates (>*φ*_gel_) demonstrating less frequency dependency, as polymer relaxation kinetics increase, from resulting confinement.^[^ ^56^, ^59^ ^]^

These key rheological, soft‐particle glass properties are what drive the material to be so versatile within regenerative medicine. The sliding nature of particle‐past‐particle at large strains not only prevents the formation of debris‐based impurities associated with quiescent gel break‐up, but also largely provides the high retention times associated with such materials. It is proposed that under dynamic oscillatory movement, typically associated with articulated regions of the body, yielding in soft‐glasses occurs as particles begin to “squeeze” past each other.^[^ ^62^ ^]^ However, as particles are able to interact within a cage‐like surrounding (a single particle surrounded by immediate neighbours), complete fracturing is prevented, forming a continuous flowing network.^[^ ^62^ ^]^ It is likely that these interactions and caging are what prevent expulsion between surfaces during manipulation, resulting in an “elastic‐like” fluid. To this end, the complex microstructure formed as a consequence to the bottom‐up approach accounts for the ability to provide structure at relatively low particle volume fractions, not always obtainable from top‐down or templating techniques.^[^ ^24^, ^63^, ^64^, ^65^, ^66^, ^67^ ^]^ Such systems, where solid‐like behaviors are not dependent on forming a “jammed” system hold a potential for improved integration, as a more open structure is less impeding to biological entities. Therefore, such matrices provide the perfect environment to offer extended therapeutic delivery under dynamic conditions.

Bespoke cellular microenvironments were fabricated through the functionalization of SyMGel particles using exemplar bio‐macromolecules relevant to cartilage tissue engineering: providing a mechanism to deliver previously rapidly cleared (low viscosity) therapies, to dynamic anatomic areas. A Michael‐type reaction was used to covalently decorate the particles, taking advantage of the unreacted acrylate groups that consequently remained due to the shear processing.^[^ ^21^, ^42^ ^]^ The simple nature of such click‐chemistry provided numerous advantages including compatibility with sensitive biological entities, removal of harsh chemicals and simplified processes, all of which greatly enhance the potential of translation towards clinic. Unfortunately, disparity between particle coating was observed: possibly due to the high surface area of a particulate system; poor mixing throughout the functionalization step, with glassy “caging” preventing long range diffusion; and/or variation in particle surface properties, as polymer termination differs dependent on the potential mechanism of formation. The latter explanation seems to hold true for SyMGels prepared at higher processing rates, where no fibronectin attachment could be observed using immunohistochemical imaging.

Cellular microenvironments are critical to biological processes throughout the human body, with cell response and fate often governed by the intrinsic chemical, mechanical and biological cues provided by their surrounding substrates.^[^ ^68^, ^69^, ^70^, ^71^ ^]^ The ability to mimic such stimuli provides the regenerative medicine toolbox with a means to active both exogenous (if being implanted) and endogenous cells, in a way that promotes improved and integrated healing, by triggering native cellular pathways. Once functionalized, SyMGels highlighted such a capacity, demonstrating a propensity to drive cellular function. Chondrocytes provided a prime example to demonstrate this, as loss of their native environment quickly effects their downstream signalling:^[^ ^72^, ^73^ ^]^ ultimately driving the production of fibrocartilage (inferior tissue matrix) instead of the clinically relevant hyaline cartilage found within articulated regions of the joint. To this end, FN‐SyMGels were able to maintain cell phenotype, preventing the regression from spheroidal (typical of native chondrocytes) to spindle‐like morphology, often a consequence to 2D culture.^[^ ^74^, ^75^ ^]^ Moreover, a high level of control over gene expression was achieved through the interplay between decoration of the gelled particles and simulating the release of cytokines (addition of chondrogenic media); demonstrating the ability to either up/down regulate signalling pertinent to proteoglycan formation, and essential to the proper load‐bearing function of articular cartilage.^[^ ^76^, ^77^ ^]^ This result was not isolated to fibronectin, where other molecules including hyaluronic acid and Wnt‐3A, also demonstrated the ability to switch on specific signalling pathways; again, all directly associated with the formation of hyaline cartilage.^[^ ^78^, ^79^, ^80^, ^81^, ^82^, ^83^ ^]^ Indeed, previous reports have shown that attachment of Wnt‐3A to surfaces can influence asymmetric division and proliferation of stem cells.^[^ ^84^, ^85^ ^]^ This work demonstrated similar properties, suggesting that such processes can be maintained using our SyMGel approach, aligning better with injectable cell therapeutic deliveries. It is believed that chemically binding the bio‐molecules to the particle interface presents a way of locally targeting the cells, whilst maintaining mechanical support. It is therefore suggested, that as the exemplar molecules used in this study did not lose their characteristic functions, functionalising is not restricted to osteo‐chondral applications, but could be used as a platform technology.

Ultimately, the simple chemical framework of the SyMGels facilitated an ease of functionalization, previously not achievable with the complex sugar chains used to fabricate current fluid gels. As such, these materials have the potential to be implanted in a minimally invasive way, to highly challenging areas of the body; for example, dynamic regions such as articulated joints, where large strains can result in either breakdown of solid (hydrogel) structures, or rapid clearance of liquid therapies. Therefore, SyMGels could provide a highly versatile platform in advanced therapies, where careful control over the decorating molecule, provides the ability to drive proper healing processes.

## 4 Conclusion

This study demonstrates the first report of fluid gels fabricated using synthetic precursors (SyMGels). The formation of such materials followed a semi‐typical photo‐radical polymerisation process, however, instead of becoming terminated by “capping”, growth of the gelled particles is controllable by the shear‐processing. Characterization of these systems has, for the first time, been able to demonstrate the complex microstructure of fluid gels; analogous to semi‐crystalline systems with gelled particles suspended between free, amorphous‐like, polymer within the continuous medium. Such structuring drives the mechanical properties, leading to behaviors akin to soft colloidal/particle glasses, with rheology highly dependent on the processing upon fabrication. Ultimately, mechanical behaviors were governed by the phase volume occupied by the particles, where gelation at higher shear resulted in less dense packing, and thus a higher degree of freedom within cages formed by neighbouring particles. Cytocompatibility was also observed to be a function of the processing, again, where more non‐gelled polymer remained, cell viability was lower. Particle functionalization with exemplar bio‐molecules, fibronectin/Hyaluronic acid/Wnt‐3A, led to enhanced cell responses, demonstrating signs of adherence, and importantly, being able to control signalling pathways conducive to healing. As such, these systems present a versatile material with the capacity to create cellular microenvironments with both mechanical support and biological cues. Ultimately, systems have the capacity to be retained in highly manipulated regions of the body, offering the potential to deliver therapeutics or provide a dynamic scaffold for cell infiltration, and facilitate native tissue repair.

## 5 Experimental Section

### Materials

Poly(ethylene glycol) diacrylate (*M*_n_ 700) (Sigma Aldrich), phosphate buffered saline (Sigma Aldrich), fibronectin (Sigma Aldrich), recombinant mouse Wnt‐3a protein (R&D Systems), HyStem thiolated hyaluronic acid (Sigma Aldrich), 1‐[4‐(2‐Hydroxyethoxy)‐phenyl]‐2‐hydroxy‐2‐methyl‐1‐propane‐1‐one (Irgacure 2959) (Sigma Aldrich), 2‐hydroxy‐2‐methyl‐1‐phenylpropanone (Omnirad 1173) (IMG Resins), Rhodamine 6G, 1% bovine serum albumin (BSA) (Cell Signalling Technology), anti‐fibronectin anti‐body (Abcam 32419), Goat Anti‐Rabbit antibody FITC (Bertin Pharma), PrestoBlue (Invitrogen), NucBlue (Invitrogen), TRI Reagent (Sigma Aldrich), High Capacity cDNA Reverse Transcription Kit (Applied Biosystems), SYBR Green PCR Mastermix (Applied Biosystems), QuaniTect Primer Assays for human *GAPDH1* and *SOX9* (Qiagen).

### Fabrication of Synthetic Microgel Suspensions (SyMGels)

Synthetic microgel suspensions (SyMGels) were prepared by first diluting poly(ethylene glycol) diacrylate (*M*_n_ 700) (PEGDA) in phosphate buffered saline (PBS), resulting in a 3% (v/v) solution. After transferring to a cell stirrer flask, the solution was mixed and UV initiator, 2‐hydroxy‐2‐methyl‐1‐phenylpropanone (Omnirad 1173), added at a final concentration of 0.1% (v/v) and allowed to mix for 30 s. Once mixed, stirring was set to either 300, 400, 500, 600, or 700 rpm. An OmniCure s2000 (Lumen Dynamics Group Inc., Canada) fitted with 5 mm light guide and standard filter (320–500 nm) was placed in the top of the stirrer flask and used to irradiate the mixture for 4 min. A Veho USB microscope was used to record changes in liquid height throughout the curing process. Following irradiation, mixing was continued for a further 30 s to prevent any residual curing in the absence of shear. Samples were then stored at 4 °C until further use.

Functionalized SyMGels (FN‐SyMGels) were prepared by first preparing the gel as described above, and subsequently mixing with an excess of fibronectin (100 µg mL^−1^), Wnt‐3a (400 ng mL^−1^), or thiolated hyaluronic acid (100 µg mL^−1^) at pH 8. Systems were then warmed at 40 °C for 1 h in a water bath allowing the additive and gel to react, before washing (via centrifugation) with PBS and stored at 4 °C until further use.

### Video Analysis of the Curing Process

Material changes throughout curing were ascertained using video analysis in MATLAB (MathWorks). In brief, a mask was applied to define the region of interest (cell stirrer flask). The video was divided into images based on time, 1 per second. Thresholding was then utilised to define the top of the fluid and used as a marker to track changes in height from its original position, as a function of time.

### Determining the Degree of Curing

was determined using a simple mass balance. SyMGels (0.5 mL) were centrifuged at 17 000 g for 10 min to separate the gelled particulate phase from the non‐gelled continuous medium. The mass of supernatant was recovered and weighed. The degree of gelation therefore defined as equal to the mass of the remaining gelled phase.

Particle volume fraction (*φ*_gel_) was determined using a similar method outlined by Garrec et al.^[^ ^26^ ^]^ (Equation 2):

(2) $$\varphi_{\mathrm{tot}}=\varphi_{\mathrm{gel}}\;+\left[\left(1-\varphi_{Q\mathrm{gel}}\right)-\varphi_{\mathrm{cont}.}\right]$$

(3) $$\varphi_{\mathrm{gel}}=\varphi_{\mathrm{tot}.}-\varphi_{\mathrm{cont}.}$$

where *φ* is the volume fraction of the gel (*φ*_gel_), an equivalent quiescent gel (*φ*_Qgel_), and the continuous phase (*φ*_cont._). Here, the effects of particle syneresis “(1 − *ϕ*_*Q*gel_)” were assumed negligible, providing a mass balance where the volume occupied by the gel was equal to the total volume minus the supernatant (Equation 3). Thus, the mass of the supernatant was converted to volume (density of PBS, 1.065 g cm^−3^) and subtracted from the initial sample volume of 0.5 mL.

### Particle Size Analysis

Particle size distributions were determined using static light scattering. A Malvern Mastersizer MS2000 (Malvern Panalytical, UK) equipped with Hydro SM manual small volume sample dispersion unit was used to obtain particle size distributions. The technique uses the Mie theory to calculate particle size, as such, particles were assumed to be monodisperse homogeneous spheres. Samples were prepared by diluting gel particles in distilled water (RI  =  1.33) to avoid multiple scattering.

### Microscopy

Optical/Fluorescent microscopy was undertaken on an EVOS M5000 (Invitrogen, UK) microscope for treated/non‐treated SyMGels and cells using phase contrast mode. SyMGels were first diluted in PBS at a ratio of 1:4 before applying to a standard slide with coverslip. Fibronectin functionalized particles were imaged using an immunohistochemical technique, whereby particles were treated with a regime of 1% BSA, primary anti‐fibronectin antibody and then secondary goat anti‐rabbit FITC anti‐body. Each step was divided by 1 h agitated incubation, followed by multiple washings/centrifugation (4000 g, 2 min) with PBS.

Confocal laser scanning microscopy (CLSM) was used to determine particle morphology. Particles were stained with Rhodamine 6G (0.1 mM) by mixing at room temperature for 20 min. The system was then washed through repeated mixing with PBS and centrifugation (4000 g for 30 s). Systems were subsequently diluted at a 1:4 ratio in PBS and placed between slide and coverslip. An Olympus IX81 (Olympus, UK) confocal microscope was then used to image the particles using a 543 nm laser and 1 µm spacing (z‐stack). Images were compiled using imaging software (ImageJ).

### Rheological Characterization

All rheometry was undertaken using a Kinexus Ultra^+^ (Malvern Panalytical, UK) rheometer equipped with 40 mm serrated parallel plate geometry. All tests were conducted at 20 °C, using a 2 mm gap height (due to the large particle size). In all cases, samples were loaded into the rheometer and allowed to equilibrate for 5 min prior to testing.

Amplitude sweeps were undertaken in stress control mode within the range of 0.01––100 Pa at a constant frequency of 1 Hz. Frequency dependent data was obtained using a constant stress found within the LVeR for all samples (0.04 Pa), over a frequency range of 0.01–10 Hz. Data collected at the higher frequency range was affected by geometry inertia and was, therefore, removed from the data presented.

Viscosity profiles were performed in stress‐controlled mode from 0.1 to 100 Pa over a ramp time of 1 min. For lower viscosity samples, tests were stopped once reaching the second Newtonian plateau to prevent expulsion of sample from the gap.

### Cell Extraction

All cell work was undertaken on primary ovine chondrocytes (unless stated otherwise), which were isolated from ovine articular knee cartilage (Staffordshire Meat Packers, Stoke‐on‐Trent, UK). Cartilage was removed from the upper condyles of the knee, finely diced, weighed and rinsed 3 times in a solution of 2% Penicillin Streptomycin (Gibco) in DPBS (Gibco). The tissue was then agitated using a magnetic stirrer and incubated overnight at 37 °C, 5% CO_2_ in chondrocyte isolation medium consisting of DMEM HAMS F12 (Lonza), supplemented with 2% Penicillin Streptomycin, ascorbic acid (50 µg mL^−1^) (Sigma), clostridial collagenase (1 mg/mL) (Sigma) and deoxyribonuclease (0.1 mg mL^−1^) (Sigma). Digested cartilage suspension was filtered through a 100 µm cell strainer and the supernatant was centrifuged at 600 g for 10 min. The chondrocytes were seeded at a density of 2 × 10^4^ cells cm^−2^ in DMEM (Gibco) supplemented with 10% foetal bovine serum (FBS) (Lonza), 1% L‐Glutamine (Gibco) and 1% Penicillin Streptomycin and expanded for 3–4 passages.

### Cell Culture

Chrondrocytes (2 × 10^4^ cell cm^−2^) were seeded into suspension wells (to promote migration in the presence of SyMGels) 24 hrs prior to the application of the SyMGels. Post‐treatment systems were cultured for a further 3 days before testing for metabolic activity and cell viability. HA‐modified SymGels (300 µL) were cultured in hanging culture (12‐well) inserts, with Y201 bone marrow stromal cells (150 µL; 6.66 × 10^5^ cells mL^−1^) placed atop of the gel suspension. Chondrogenic medium (DMEM supplemented with L‐ascorbic acid 2‐phosphate (50 µg mL^−1^), dexamethasone (100 nM), TGFß‐3 (10 ng mL^−1^), insulin‐transferrin‐selenium, 1% penicillin‐streptomycin and 1% L‐glutamine) was added to the well and cells were cultured for 9 days at 37 °C with 21% oxygen. Basic medium (DMEM supplemented with 10% FCS, 1% penicillin‐streptomycin and 1% L‐glutamine) and non‐HA controls were included.

### Metabolic Activity Assay

was undertaken using a PrestoBlue assay kit (Invitrogen). In brief, cells were washed once with DPBS, and 1 mL medium supplemented with 10% PrestoBlue was added to each well. Cells were then incubated for 4 hrs. 50 µL supernatant from each well was transferred to one well of a 96 well plate and fluorescence was measured using a Tecan Spark (Tecan Trading AG, CH) plate reader with excitation/emission wavelengths set at 550/620 nm.

### Live/Dead

was conducted using a ReadyProbes Cell Viability Imaging Kit (Invitrogen). The assay was conducted in accordance with the manufacturer's instructions by adding two drops of NucBlue live reagent and 2 drops of NucGreen dead reagent directly to each mL culture medium and incubating for 15 min. Cells were imaged using a fluorescent microscope equipped with 405 and 488 nm lasers.

### Wnt‐3A Luciferase Assay

was conducted using Y201 TCF/LEF reporter cells. Wnt‐modified SyMGels (70 µL) were pipetted into a black 96‐well plate. TCF/LEF luciferase reporter cell suspension (20 µL, 1.5 × 10^6^ cells mL^−1^) was added on top the gels and incubated for 24 hours. Luciferase expression was determined using the ONE‐Step Luciferase Assay System as per the manufacturer's instructions. In addition, cells were seeded into 96‐well plate and allowed to adhere overnight, prior to treatment. Wnt‐SyMGels (70 µL) were then placed over the cells and incubated for a further 48 hours. GFP expression was then determined with an EVOS fluorescent microscope.

### Gene Expression

RNA was isolated from cell‐seeded SyMGels using TRI Reagent as per the manufacturer's instructions and converted into cDNA using High Capacity cDNA Reverse Transcription Kit, again as per manufacturer's instructions. Gene expression analysis was performed using SYBR Green‐based quantitative real‐time polymerase chain reaction (qRT‐PCR) with pre‐optimised QuantiTect primer assays and an AriaMx Real‐Time PCR System (Agilent Technologies). Relative gene expressions in the form 2^−ΔCT^ were calculated and expression levels of *SOX9* were normalized to expression levels of the housekeeping gene *GAPDH1*.

### Statistical Analysis

All data presented shows the average of at least 3 repeats, with error bars showing the 95% confidence interval. Statistical significance was probed using one‐way and two‐way ANOVA and *p*‐value quoted as * *p* < 0.05, ** *p* < 0.01 and *** *p* < 0.001.

## Conflict of Interest

The authors declare no conflict of interest.

## Data Availability

Research data are not shared.
